# Mitigating Response of *SlCSE06* Induced by 2-Ethylfuran to *Botrytis cinerea* Infection

**DOI:** 10.3390/plants14040575

**Published:** 2025-02-13

**Authors:** Huilan Ye, Hongdou Gao, Jinnian Li, Linye Lu, Shilan Zheng, Chengxin Wu, Youliang Jin, Chengjuan Cao, Haisheng Zhu, Shuang Liu, Fenglin Zhong

**Affiliations:** 1 College of Horticulture, Fujian Agriculture and Forestry University, Fuzhou 350002, China; 19835620037@163.com (H.Y.); ghd1396677078@163.com (H.G.); shrryi@foxmail.com (J.L.); 13459760398@163.com (L.L.); shilan0607@163.com (S.Z.); wwxxcy1916@163.com (C.W.); jin011207@163.com (Y.J.); 17689628171@163.com (C.C.); zhs0246@163.com (H.Z.); 2 Fuzhou Smart Agriculture (Seed Industry) Industry Innovation Center, Fuzhou 350002, China; 3 Key Laboratory of Crop Biological Breeding in Fujian and Taiwan, Ministry of Agriculture and Rural Affairs, Fuzhou 350002, China

**Keywords:** tomato, caffeoyl shikimate esterase, *Botrytis cinerea*, 2-ethylfuran

## Abstract

Tomato (*Solanum lycopersicum* L.) is a major economic vegetable crop globally, yet it is prone to gray mold disease caused by *Botrytis cinerea* infection during cultivation. Caffeoyl shikimate esterase (CSE) is a crucial component of the lignin biosynthesis pathway, which significantly contributes to plant stress resistance. Therefore, investigating the expression patterns of *SlCSE* after *Botrytis cinerea* infection may offer a theoretical foundation for breeding resistant tomato varieties. In this study, 11 *SlCSE* family members were identified from the tomato genome using bioinformatics analyses. Public transcriptome databases and RT-qPCR experiments were used to analyze gene expression in tomato tissues, responses to *Botrytis cinerea* infection, and the temporal characteristics of the response to 2-ethylfuran treatment during infection. These experiments resulted in the identification of the key gene *SlCSE06*. Transgenic tomato lines that overexpressed *SlCSE06* were constructed to examine their resistance levels to gray mold disease. Many *SlCSE* genes were upregulated when tomato fruit were infected with *Botrytis cinerea* during the ripening stage. Furthermore, 24 h after treatment with 2-ethylfuran, most *SlCSE* genes exhibited increased expression levels compared with the control group, but they exhibited significantly lower levels at other time points. Thus, 2-ethylfuran treatment may enhance the responsiveness of *SlCSEs*. Based on this research, *SlCSE06* was identified as the key gene involved in the response to *Botrytis cinerea* infection. The *SlCSE06*-overexpressing (OE6) tomato plants exhibited a 197.94% increase in expression levels compared to the wild type (WT). Furthermore, the lignin content in OE6 was significantly higher than in WT, suggesting that the overexpression of *SlCSE06* enhanced lignin formation in tomato plants. At 5 days post-inoculation with *Botrytis cinerea*, the lesion diameter in OE6 decreased by 31.88% relative to the WT, whereas the lignin content increased by 370.90%. Furthermore, the expression level of *SlCSE06* was significantly upregulated, showing a 17.08-fold increase compared with the WT. These findings suggest that 2-ethylfuran enhances the activation of the critical tomato disease resistance gene *SlCSE06* in response to gray mold stress, thereby promoting lignin deposition to mitigate further infection by *Botrytis cinerea*.

## 1. Introduction

Tomato (*Solanum lycopersicum* L.) is a herbaceous plant, either annual or perennial, that belongs to the Solanaceae family and is a significant economic crop [1]. China ranks among the world’s leading tomato producers. According to FAO statistics, the area dedicated to tomato cultivation in China reached 1.1417 million hectares in 2022, yielding approximately 68.34 million tons of fruit. However, tomatoes are susceptible to diseases that significantly affect economic returns. Among these diseases, gray mold, caused by *Botrytis cinerea* [2], is a prevalent fungal infection that hinders tomato growth and development. This disease is challenging to prevent during the post-harvest storage of tomatoes [3]. A shift in consumer health consciousness has highlighted the limitations of existing physical and chemical methods for controlling *Botrytis cinerea.* Safe, environmentally friendly, and efficient bioactive substances represent a research focal point and are considered potential alternatives. Specifically, biocompatible plant volatile organic compounds (VOCs) have a wide range of applications in pathogen defense. Tomatoes are rich in flavor compounds, including reducing sugars, organic acids, free amino acids, and VOCs, which contribute to their unique aroma. These compounds can directly combat pathogens through cytotoxic effects and indirectly regulate them by stimulating host immune responses [4]. 2-Ethylfuran, a volatile furan compound found in tomato, has significant antibacterial properties [5]. Investigating the theoretical mechanisms underlying 2-ethylfuran’s effectiveness in increasing tomato resistance to *Botrytis cinerea* will establish a foundation for the further exploration of plant VOC applications in the management of pathogen-related stress.

*Botrytis cinerea* simultaneously secretes a range of cell wall-degrading enzymes, including cellulase, pectinase, hemicellulase, keratinase, and protease, and the degraded plant cell wall components act as carbon sources for *Botrytis cinerea* growth [6,7]. In response, plant tissues employ a specific recognition system, along with fundamental defense responses, to thwart *Botrytis cinerea*’s infiltration and restrict its growth. These defenses include processes such as cell death, defense gene expression, antifungal compound synthesis, callose deposition, and cell wall lignification [8]. Lignin, a pivotal product of the phenylpropanoid biosynthesis pathway, is a vital component of the cell wall and plays a critical role in plant resistance to pathogenic stress [9]. Lignin is a complex polymer composed of phenolic compounds and is characterized by a highly intricate biosynthetic network. In total, 14 enzymes, including cinnamate-4-hydroxylase, hydroxycinnamoyl-CoA shikimate/quinate hydroxycinnamoyltransferase (HCT), peroxidase, and laccase (LAC), are essential for lignin monomer biosynthesis [10]. In the lignin biosynthesis pathway, coenzyme A is converted to p-coumaroyl shikimate by HCT. This compound is then converted to caffeoyl shikimate through the action of cinnamate-3-hydroxylase, before being catalyzed into caffeoyl-CoA by HCT. Caffeoyl shikimate esterase (CSE), a key enzyme in lignin biosynthesis, was first identified in *Arabidopsis thaliana* in 2013. This pathway catalyzes the conversion of caffeoyl shikimate to caffeoyl-CoA through the action of 4-coumarate:CoA ligase, thereby bypassing a second HCT reaction [11]. The identification of this pathway enhanced the theoretical framework of lignin synthesis and was crucial for elucidating the mechanisms underlying plant lignin deposition and responses to pathogenic stress.

At present, 18, 24, 8, 2, 16, 9, 9, and 6 *CSE* genes have been identified in poplar, pear, cucumber, barrel medic, *Arabidopsis*, maize, rice, and grape [12,13,14,15]. The *CSE* genes in *Arabidopsis* [16,17], poplar [18], petunia [19], and pear have been shown to be crucial for plant and fruit lignification, phenylpropanoid metabolism, and carbohydrate synthesis. Additionally, cucumber *CSE1* [20] and *CSE5* [14] play crucial roles in resisting fungal diseases and leaf spot infections. Specifically, *CsCSE1* regulates the expression of genes related to lignin synthesis [10], including *CsLACs*, *CsCOMTs*, *CsCCRs*, and *CsCADs*, thereby mediating cucumber disease resistance. Furthermore, a silenced mutant of *CsCSE5* exhibits a marked reduction in resistance to both fungal diseases and leaf spot infections. These data have enhanced our understanding of the *CSE* family’s role in responses to disease-related stress. However, the *CSE* family has not yet been identified in tomato. Here, the expression of specificity of tomato *SlCSEs* in response to *Botrytis cinerea* infection-related stress across different tissues was investigated. Additionally, bioinformatics-based sequence characteristics of the gene family were analyzed and the expression patterns of *SlCSE* genes in response to *Botrytis cinerea* infection-related stress and 2-ethylfuran treatments were examined. Key genes were selected for functional validation, establishing a foundation for future studies on the functions of this gene family and offering a theoretical basis for breeding disease-resistant tomato varieties.

## 2. Results

### 2.1. The Identification, Chromosomal Distribution, and Physicochemical Properties of the SlCSE Family Members

**Figure 1:**
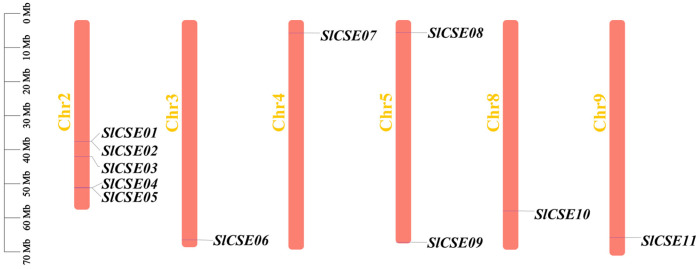
Chromosomal localization of the *SlCSE* family members.

In total, 11 *SlCSE* family members, spread across six chromosomes, were identified in the tomato genome (Figure 1). Based on their chromosomal positions, the 11 genes were designated as *SlCSE01–11*. Among the chromosomes, Chr2 was the shortest; however, it had the highest distribution of *SlCSE* genes, with five members (45.45%). Chr3, 4, 5, 8, and 9 each contained a single *SlCSE* gene. The number of amino acids in the SlCSE proteins ranged from 178 (*SlCSE04*) to 404 (*SlCSE10*). The molecular weights of the SlCSE proteins varied from 20.38 kDa (*SlCSE04*) to 45.16 kDa (*SlCSE10*), whereas the isoelectric point ranged from 5.8 (*SlCSE05*) to 9.17 (*SlCSE02*). There were four acidic and seven basic proteins. Six *SlCSEs* exhibited an instability coefficient of less than 40, indicating that they are stable. The GRAVY values were below zero, indicating that all of the SlCSE proteins are hydrophilic. Additionally, the subcellular localization analysis revealed that five *SlCSE* genes are located in the cytoplasm, three in chloroplasts, and the remaining three are distributed among the cytoskeleton, mitochondria, and nucleus. Furthermore, all the *SlCSEs* transmembrane regions are situated outside the cell and lacked signal peptides (Table 1).

### 2.2. Proteins Encoded by the SlCSE Family Members

**Figure 2:**
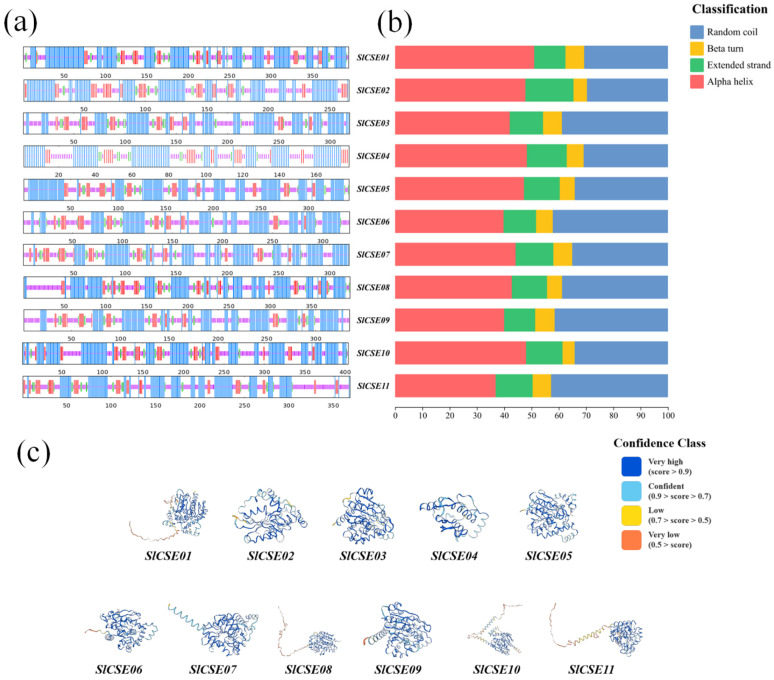
The secondary and tertiary structures of *SlCSE* family member proteins are presented. (**a**–**c**): Analysis of the secondary (**a**,**b**) and tertiary (**c**) structures of the SlCSE proteins.

The analysis of the spatial protein structures of *SlCSEs* revealed that there are four types of the secondary structures among the family members (Figure 2a,b). Among these, α-helix and random coil represented a relatively high proportion, whereas β-turn and extended chain constituted a smaller fraction. Following the modeling and visualization of the tertiary protein structures of the *SlCSE* family members (Figure 2c), it was determined that *SlCSE01* and *SlCSE02* utilize the protein structure of Caffeoylshikimate esterase, identified by the ID number A0A314L9X9.1.A, as their reference template. In contrast, *SlCSE03* adopts the structure of the Serine aminopeptidase S33 domain-containing protein, carrying the ID number A0A3Q7F1M4.1.A, as its reference model. Additionally, *SlCSE04* and *SlCSE05* share the same reference template, which is the protein structure of Caffeoylshikimate esterase-like, identified by the ID number A0A1U7V8R7.1.A. Meanwhile, *SlCSE06*, *SlCSE07*, and *SlCSE08* each draw upon unique reference models: the structures of Serine aminopeptidase S33 domain-containing proteins with the ID numbers A0A3Q7FUU7.1.A, A0A4S4F2I2.1.A, and A0A5N6RME6.1.A, respectively. Furthermore, *SlCSE09* and *SlCSE10* use the protein structures of Caffeoylshikimate esterase, identified by the ID numbers A0A1U7WY18.1.A and A0A1J6KRU2.1.A, respectively, as their reference templates. Lastly, *SlCSE11* adopts the structure of the Serine aminopeptidase S33 domain-containing protein, identified by the ID number A0A5J5UMI0.1.A, as its reference model.

### 2.3. Phylogenetic and Evolutionary Analyses of the CSE Family

**Figure 3:**
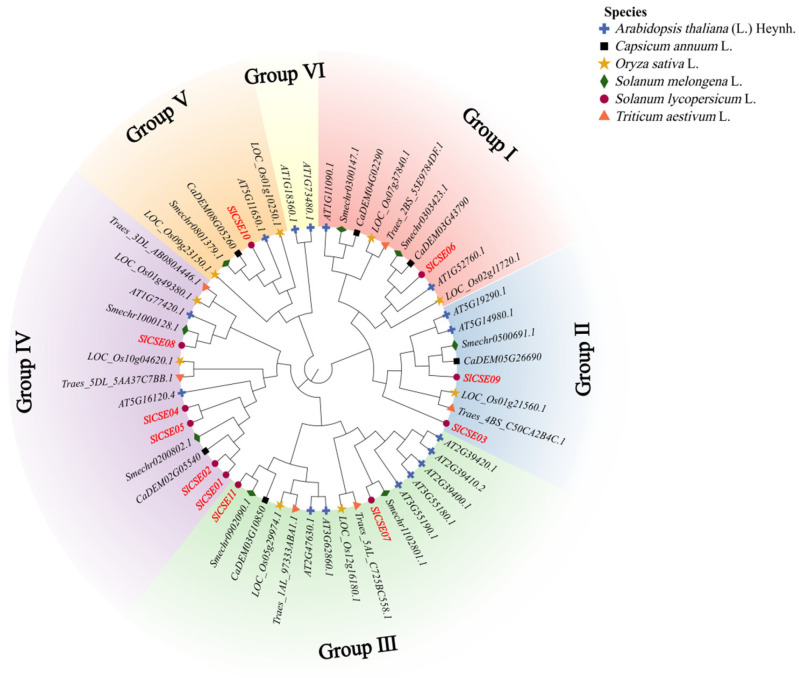
Rootless phylogenetic trees depicting the *CSE* gene families of six species—tomato, eggplant, pepper, *Arabidopsis*, rice, and wheat—were illustrated using circles, diamonds, squares, plus signs, stars, and triangles, respectively. Different color patches indicate distinct subfamilies.

To elucidate the biological characteristics of, and genetic relationships among, *CSE* gene families across different species, the amino acid sequences of 16, 9, 6, 8, 6, and 11 *SlCSEs* were selected from *Arabidopsis*, rice, wheat, eggplant, pepper, and tomato using MEGA11 software (version 11.0.13) for multi-sequence alignment and phylogenetic tree construction (Figure 3). The *CSE* family members from these six species were categorized into six subfamilies. Notably, tomato *CSEs* were distributed among subfamilies I–V, with the most members being in subgroup IV. Additionally, *SlCSE01* and *SlCSE02*, as well as *SlCSE04* and *SlCSE05*, demonstrated a close homology, as did *SlCSE08* and Smechr1000128.1. From an evolutionary perspective, *CSE* family members from rice and wheat, as well as those from tomato, eggplant, and pepper, were closely related. This suggests that they share similar botanical functions and possess traits conserved throughout evolution. Taxonomically, significant differences existed among the *CSE* family members of Solanaceae, Cruciferae, and Poaceae. This suggests that unique genetic traits have emerged during the evolutionary process.

### 2.4. Gene Structure and Conserved Protein Motifs and Promoter Cis-Acting Elements in the SlCSE Family Members

**Figure 4:**
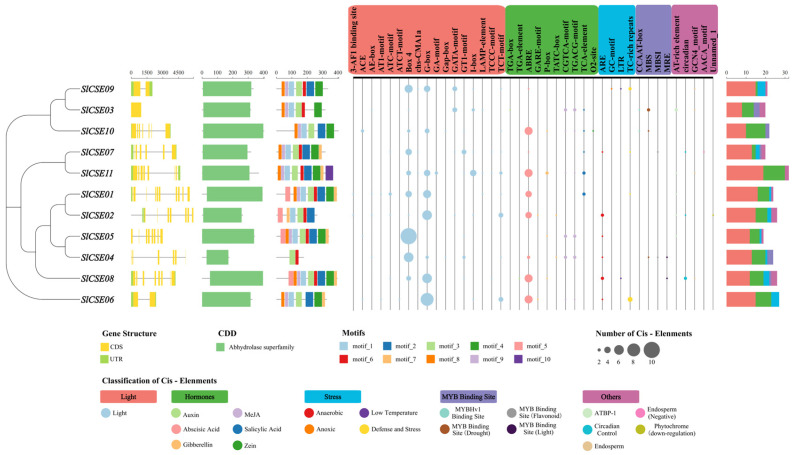
Phylogenetic tree, gene structure, conserved domain, conserved motifs, and promoter cis-acting element analyses of the *SlCSE* family members.

To further investigate the structure of the *SlCSE* family, we analyzed the phylogenetic relationships, exon–intron structures, conserved protein motifs, and overall structures of *SlCSEs* (Figure 4). The tomato *SlCSEs* were categorized into five distinct groups, aligning with the results of the phylogenetic and evolutionary analyses of the *CSE* family. The exon–intron structural analysis revealed that the *SlCSEs* typically contained an average of 5.96 exons (predominantly 6–8) and 4.96 introns (mainly 5–7).

Additionally, the conserved protein domain analysis revealed that *SlCSEs* possessed conserved abhydrolase domains. An analysis of the conserved protein motifs in *SlCSEs* using MEME indicated that all the *SlCSEs* included motif 3 and generally contained motifs 1, 2, 4, 6, 8, and 9. Furthermore, motif 10 was exclusively present in *SlCSE11*, whereas motif 5 was found only in *SlCSE01*, *SlCSE02*, *SlCSE05*, and *SlCSE08*. This suggests that motif 5 is a crucial conserved protein motif among proteins in this group and other groups. Moreover, motif 3 was present in all the *SlCSEs*, demonstrating high conservation.

To further investigate the differences in promoter function among *SlCSEs*, the promoter region sequences located 2000 bp upstream of the start codon of family members were extracted for cis-acting element analysis. The analysis identified 40 cis-acting elements, 17 related to light responses, 10 related to hormone responses, 4 associated with adverse stresses, 4 MYB binding sites, and 5 other functionally related elements. Additionally, *SlCSEs* contained numerous photoresponsive elements, such as Box4 and G-box, alongside abscisic acid-responsive elements (ABREs). Specifically, *SlCSE05–07* exhibited the highest number of Box4, G-box, and ABRE components, respectively. These findings suggest that *SlCSEs* are highly conserved through evolution and play crucial roles in tomato growth and development through light, hormone signaling, MYB transcriptional regulation, and stress-response networks.

### 2.5. Collinearity and Selection Pressure Analyses of the CSE Families

**Figure 5:**
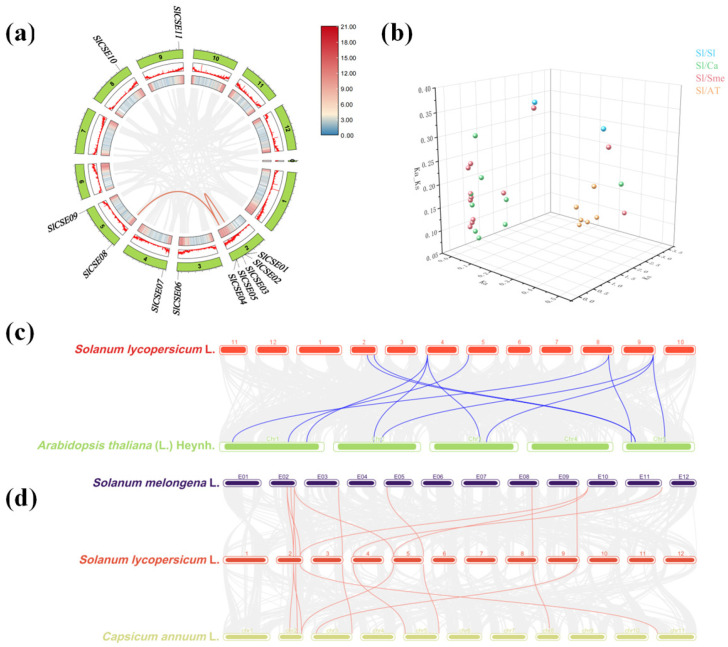
Analysis of collinearity and selection pressure within the *CSE* families. (**a**) *SlCSE* distribution and segmental replication event analysis. In the green panel, Chromosomes 1–12 correspond to tomato chromosomes, the red line represents collinear gene pairs, and the gray area delineates the collinear regions. (**b**) Selection stress analysis. Blue, green, red, and orange circles represent tomato, tomato/pepper, tomato/eggplant, and tomato/*Arabidopsis* collinear *CSE* gene pairs, respectively. (**c**) Collinearity analysis between tomato and *Arabidopsis*. The gray line in the background denotes the collinearity blocks in the tomato and *Arabidopsis* genomes, and the blue line highlights homologous *CSE* gene pairs. (**d**) Collinearity analysis among tomato, eggplant, and pepper. The gray line in the background represents the collinearity block in the tomato, eggplant, and pepper genomes, and the red line illustrates the homologous *CSE* gene pairs.

As shown in Figure 5a, there were two distinct replication events within the *SlCSE* family. *SlCSE01* and *SlCSE04* represent a tandem replication. In contrast, *SlCSE04* and *SlCSE08* represent chromosomal fragment replication. To further elucidate the evolutionary mechanisms that affected the *SlCSE* family, a collinearity analysis was conducted using MCScanX that analyzed members of the *CSE* families across tomato, pepper, eggplant, and *Arabidopsis* (Figure 5c,d). The results revealed 10 collinear relationships between nine *SlCSEs* and nine *CaCSEs*. Additionally, 12 collinear relationships were identified between nine *SlCSEs* and nine *SmCSEs*. Furthermore, seven collinear relationships were found between five *SlCSEs* and six *AtCSEs*.

To understand the selective pressures and evolutionary rates of the *SlCSE* family, the non-synonymous replacement rate (Ka), synonymous replacement rate (Ks), and Ka/Ks ratios for replication events involving tomato/tomato, tomato/pepper, tomato/eggplant, and tomato/*Arabidopsis* were calculated (Figure 5b). After excluding gene pairs that had significant sequence differences, the resulting data presented in the figure indicate that all the gene pairs exhibited 0 < Ka/Ks < 1, suggesting that they underwent purifying selection during evolution.

### 2.6. Tomato Transcription Factor and CSE Family Regulatory Network

**Figure 6:**
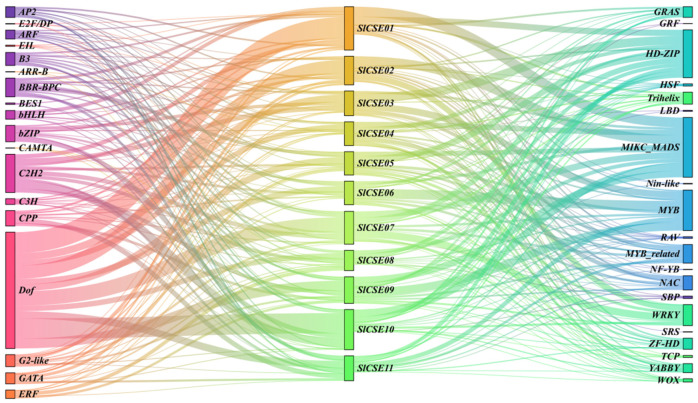
Prediction of upstream transcription factors regulating the *SlCSE* family.

The Fimo online platform was used to investigate the regulatory relationships between tomato transcription factors and the *SlCSE* family (Figure 6). The analysis revealed 6957 pairs, comprising 565 transcription factors and 11 *CSEs* in tomato. Notably, a number of transcription factor families, including Dof, MIKC_MADS, HD-ZIP, MYB, and C2H2, were involved in regulating *SlCSEs*. These findings indicate that the *SlCSE* family is primarily regulated by transcription factors associated with growth, development, and stress responses.

### 2.7. Analysis of the Expression Characteristics of SlCSE Family Members

#### 2.7.1. Expression Analysis of the *SlCSE* Family Members Across Various Tissues

**Figure 7:**
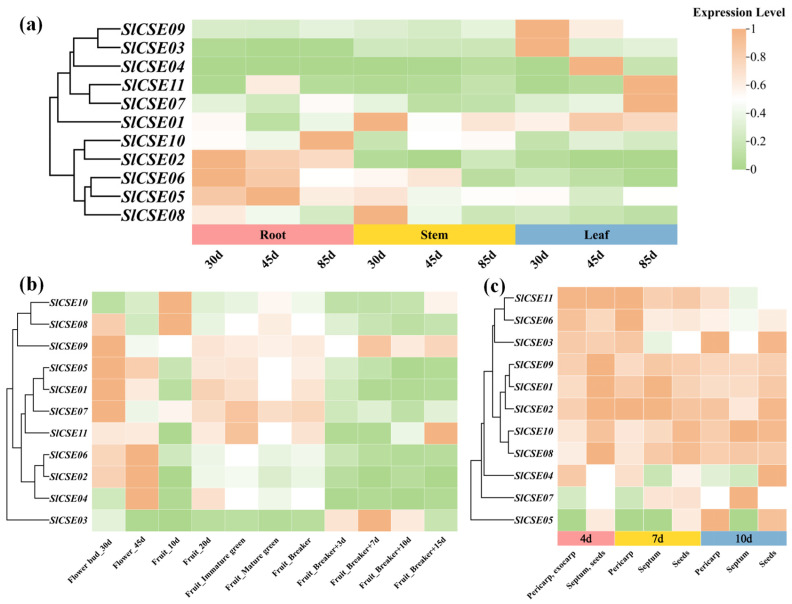
Analysis of *SlCSE* family members expression in tissues. (**a**) Expression patterns in vegetative organs; (**b**) expression profiles during flower and fruit growth and development; and (**c**) expression dynamics during various fruit tissue developmental stages.

Utilizing the ‘MicroTom’ expression database (https://eplant.njau.edu.cn/microTomBase/) (accessed on 5 August 2024) and the tomato functional genome database (http://ted.bti.cornell.edu/cgi-bin/TFGD/digital/home.cgi) (accessed on 5 August 2024), the transcriptome sequencing results from various tomato tissues and fruit were screened. The expression levels of the *SlCSE* family members across different tissues were analyzed (Figure 7a,b). *SlCSEs* were categorized into two groups within vegetative organs, with higher expression levels observed in the leaves, roots, and stems. Furthermore, the expression levels of certain *SlCSEs* varied with seedling age. For instance, *SlCSE07* and *SlCSE11* exhibited higher expression levels in tomato leaves in 85-day-old seedlings. *SlCSE08* was exclusively expressed in tomato stems of 30-day-old seedlings. In reproductive organs, *SlCSEs* were classified into four groups, with expression levels specifically upregulated during the flower bud, flower, green maturity, and color-breaking developmental stages, respectively. Specifically, *SlCSE01*, *SlCSE05*, *SlCSE07*, and *SlCSE09* had the highest expressions in flower buds, whereas *SlCSE02*, *SlCSE04*, and *SlCSE06* exhibited the highest expressions in flowers. Additionally, *SlCSEs* were classified into five groups based on expression in different tomato fruit tissues. On day 4 post-pollination, all the *SlCSEs*, except *SlCSE03*, *SlCSE04*, and *SlCSE06*, exhibited increased expression levels in the separator and seeds. On day 7 post-pollination, the expression levels of *SlCSE02*, *SlCSE03*, *SlCSE06*, and *SlCSE11* were elevated in the peel. The expression levels of *SlCSE01* and *SlCSE09* were high in the separator. The remaining *SlCSEs* exhibited higher expression levels in the seeds. On day 10 post-pollination, *SlCSE05* and *SlCSE11* exhibited increased expression levels in the pericarp. The expression levels of *SlCSE07*, *SlCSE08*, and *SlCSE10* were elevated in the separator. The remaining *SlCSEs* exhibited higher expression levels in seeds. Additionally, *SlCSE07* and *SlCSE10* demonstrated significantly increased expression levels in the peel at 10 days post-pollination compared with the septum and the seeds.

#### 2.7.2. Effects of 2-Ethylfuran Treatments on *SlCSE* Family Members Expression Profiles During Post-Harvest *Botrytis cinerea* Infection

**Figure 8:**
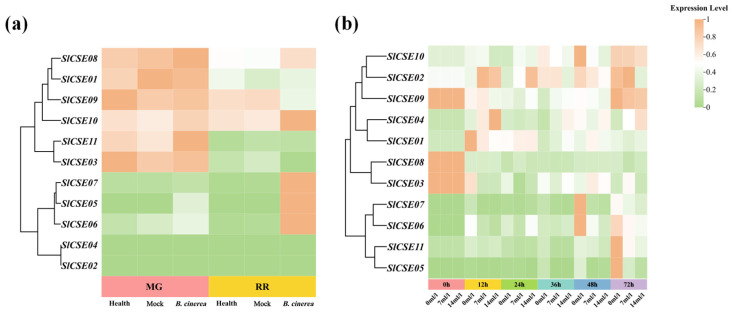
Correlation analysis of the post-harvest responses of the *SlCSE* family members to *Botrytis cinerea* infection. (**a**) Expression analysis of the mature green (MG) and red ripe (RR) fruits under health (normal), mock (inoculated with sterile water), and *Botrytis cinerea* (inoculated with *Botrytis cinerea*) treatments; (**b**) expression analysis of *Botrytis cinerea* infection with varying concentrations of 2-ethylfuran (0 mL/L, 7 mL/L, and 14 mL/L).

To investigate the expression of *SlCSE* family members during post-harvest *Botrytis cinerea* infection, public transcriptome data (PRJNA762085) obtained from the NCBI database (https://www.ncbi.nlm.nih.gov/) (accessed on 5 August 2024) were analyzed (Figure 8a). The expression levels of *SlCSE* family members were classified into two groups based on treatments: post-harvest normal and punctured plus *Botrytis cinerea* infection. Notably, the expression levels of *SlCSE05–08* and *SlCSE10* in tomato fruit at the red ripening stage were significantly upregulated following infection with *Botrytis cinerea*.

This study aimed to further investigate the roles of *SlCSEs* in the response to 2-ethylfuran treatment during *Botrytis cinerea* infections of post-harvest tomatoes. The relative expression levels of *SlCSEs* were quantified using RT-qPCR (Figure 8b). The expression levels of *SlCSEs* were classified into four distinct groups, with most genes exhibiting a temporal response characterized by an ‘increase–decrease–increase’ pattern under each treatment. Furthermore, at 24 h post-2-ethylfuran treatment, the expression levels of all the *SlCSEs*, except *SlCSE03* and *SlCSE11*, were significantly higher than those of the control at the same time point. At the remaining time points, the 2-ethylfuran treatments consistently reduced the expression levels of *SlCSEs*. Additionally, treatment with 7 mL/L of 2-ethylfuran prolonged the duration of increased expression levels of *SlCSE03*, *SlCSE05*, and *SlCSE07*, while reducing the duration of the decreased expression level of *SlCSE06*.

The preliminary results indicated that nine *SlCSEs* were upregulated within 24 h after 2-ethylfuran treatments, enhancing resistance to *Botrytis cinerea* in post-harvest tomatoes. Additionally, *SlCSE05–07* played crucial roles in the response to *Botrytis cinerea*. Among them, *SlCSE06* exhibited the highest efficacy in inhibiting gray mold induced by 2-ethylfuran.

### 2.8. SlCSE06 Cloning and the Establishment of OE6 Tomato Plants

**Figure 9:**
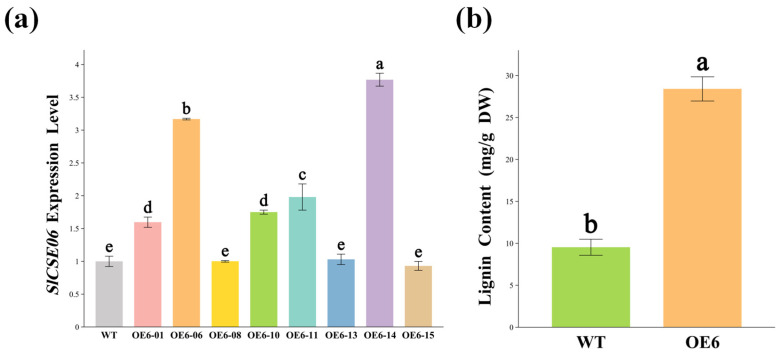
RT-qPCR and lignin content detection in OE6 positive plants. Measurements of (**a**) the relative *SlCSE06* expression levels and (**b**) the lignin content. The lowercase letters in the figures indicate saliency at *p* < 0.05.

To further investigate the biological function of *SlCSE06*, *SlCSE06*-overexpressing (OE6) tomato plants were constructed using a tomato genetic transformation system. Actin was used as an internal reference gene, and the *SlCSE06* expression levels in OE6 and WT plants were detected using RT-qPCR (Figure 9a). The *SlCSE06* expression levels in OE6-01, OE6-06, OE6-10, OE6-11, and OE6-14 were significantly higher than those in the WT, with increases of 59.65%, 216.84%, 74.99%, 98.01%, and 276.88%, respectively. Therefore, the OE6-01, OE6-06, OE6-10, OE6-11, and OE6-14 plants, which exhibited higher expression levels of *SlCSE06*, were selected for further analysis. The lignin content in mature OE leaves was 197.94% higher than that in mature WT leaves, suggesting that the overexpression of *SlCSE06* promoted lignin synthesis in tomato plants.

### 2.9. The SlCSE06 Expression Pattern in Response to Botrytis cinerea Infection

**Figure 10:**
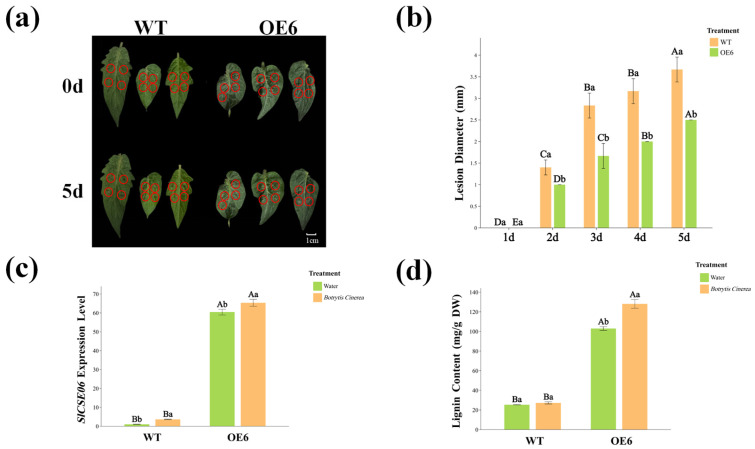
Response patterns of WT and OE6-positive tomato plants to *Botrytis cinerea* infection. (**a**) Leaf lesion morphology; (**b**) changes in lesion diameter over time; (**c**) characteristics of the *SlCSE06* response; and (**d**) lignin content. In the figure, the red circles pinpoint the location of the plaque. Uppercase letters denote the statistical significance of the same treatment across different time points, with *p* < 0.05. Meanwhile, lowercase letters indicate the statistical significance of the same treatment at varying time points, also using *p* < 0.05.

To further explore the response of *SlCSE06* to *Botrytis cinerea* infections in tomato plants, mature leaves from the WT and OE6 were collected and subjected to treatment with either distilled water (control) or a *Botrytis cinerea* suspension. Disease symptoms, lignin contents, and *SlCSE06* expression patterns under *Botrytis cinerea* infection-related stress were analyzed, as illustrated in Figure 10a,b. At 5 days post-inoculation with *Botrytis cinerea*, OE6 leaves showed enhanced resistance to *Botrytis cinerea* by exhibiting reduced damage at the infection sites. Additionally, the diameters of the fungal lesions on OE6 leaves were significantly smaller than those on WT leaves by 31.88%. These findings suggest that OE6 exhibits an enhanced ability to withstand *Botrytis cinerea* infection-related stress.

Furthermore, the relative *SlCSE06* expression levels and lignin contents in tomato leaves infected with *Botrytis cinerea* for 5 days were measured and analyzed (Figure 10c,d). The expression level of *SlCSE06* under *Botrytis cinerea* infection-related stress increased by 261.59% in the WT but only 8.18% in OE6 compared with the distilled water treatment. Both values were significantly higher than the control. Additionally, after *Botrytis cinerea* infection, the *SlCSE06* expression level in OE6 was 17.08 times higher than in the WT, indicating heightened responsiveness. The trend in lignin content closely mirrored that of *SlCSE06* expression. In WT and OE6, the lignin content after *Botrytis cinerea* infection increased by 7.14% and 24.34%, respectively, compared with the distilled water treatment. Additionally, OE6 exhibited a greater lignin deposition under *Botrytis cinerea* infection-related stress, with a remarkable increase of 370.90%, which significantly exceeded that of the WT.

These results indicate that *SlCSE06* overexpression enhances tomato resistance to *Botrytis cinerea* infection by activating its own expression, which promotes lignin accumulation in response to *Botrytis cinerea* infection-related stress.

## 3. Discussion

In the lignin biosynthesis pathway, *CSE* is the crucial enzyme that converts caffeoyl shikimic acid into both caffeic acid and shikimic acid [14,16,19]. It has been identified across various plant species [12,13,14,15]. In the *Arabidopsis cse-2* loss-of-function mutant, the lignin content is reduced compared with the WT. This decrease, coupled with a fourfold increase in the conversion rate of cellulose to glucose [21], results in vascular bundle shrinkage and impairs plant growth and development [17]. In pear, *CSE1* is significantly associated with lignin deposition and stone cell development [13]. Petunia *ir-CSE* plants show reduced numbers of xylem layers, which negatively affects their growth [19]. Additionally, employing the caffeoyl shikimic acid pathway can enhance the expression of genes associated with volatile compounds, such as cinnamate-3-hydroxylase, HCT, and 4-coumarate:CoA ligase. Consequently, this leads to reduced levels of various floral volatiles, including phenylalanine and caffeic acid. *EuCSE4* is upregulated during leaf growth and development, suggesting its potential role in the regulatory synthesis of Oenothein B in leaves [22]. In poplar, *CSE2* and *CSE12* may also play roles in stress responses related to cell wall biosynthesis [12]. Silencing *CSE5* in cucumber results in reduced resistance to *Podosphaera xanthii* and *Corynespora cassiicola* [14]. However, systematic identification and related studies on the *SlCSE* family are currently lacking.

This study identified 11 *SlCSEs* in the tomato genome, which is a different number of members than present in other species. The *Arabidopsis* genome contains 16 family members, whereas the cucumber, poplar, and pear genomes contain 8, 18, and 24 family members, respectively. Despite the different numbers of members, all the encoded *CSEs* exhibit highly conserved structural domains. An analysis of the physicochemical properties of the *SlCSEs* revealed similarities among members. They were all hydrophilic proteins predominantly localized in the cytoplasm and chloroplasts. This suggests that the physiological functions of tomato are closely related. The 11 members of the *SlCSE* family were classified into five subfamilies, with subfamily IV having the highest number of members (5, 45.45%). A structural analysis of the encoded proteins indicated that the secondary structures were primarily α-helices and random coils, which were represented by nine distinct protein structural models. Furthermore, a gene structure analysis demonstrated that all the *SlCSEs* possessed a conserved abhydrolase structural domain [14] and a motif associated with the N-myristoyl transferase signal at the second position, suggesting a strong correlation between *SlCSEs* and protein N-myristoylation [23]. Additionally, a motif linked to the active sites of glucose-6-phosphate dehydrogenase was unique to subfamily IV. Thus, this subfamily may play roles in stress resistance and morphological development [24]. The Ka/Ks ratios of collinear gene pairs within tomatoes and across various species were consistently below 1, indicating that these genes have experienced purifying selective pressure throughout evolution. These findings suggest that *SlCSEs* have remained conserved during evolution, preserving essential functions while also exhibiting functional differences across various subfamilies. An analysis of the promoter region, located 2000 bp upstream of the coding sequence, revealed that the *SlCSEs* contain numerous light-responsive elements, including Box4 and G-box, as well as ABREs. However, AT-rich and circadian elements were identified in only a small number of *SlCSEs*. This observation reflects both the conservation and diverse functions of *SlCSEs* throughout evolution. Moreover, this finding indicates that *SlCSEs* play a crucial role in tomato growth and development through mechanisms involving light, abscisic acid, MYB transcriptional regulation, and stress-response networks [25,26,27].

*Botrytis cinerea* infection remains a significant limiting factor in tomato production. Chemical fungicides traditionally used to combat these infections, such as dimethyldihydropyrimidine derivatives and pyrole compounds, have been gradually replaced due to application restrictions and increased *Botrytis cinerea* drug resistance. Plant VOCs, known for their biocompatibility, accessibility, and practicality, are widely used to combat pathogens and spoilage-related organisms. These compounds can directly inhibit fungal activity and enhance plant defense responses to pathogens, thereby having significant potential for practical applications. Certain VOCs can regulate intermediates and associated genes in the phenylpropanoid biosynthetic pathway, aiding in resistance to disease stress. During pathogen infection, plants detect and modulate VOCs derived from phenylpropanoid metabolism, including phenylpropanoids/benzenes and terpenoids, like (+)-limonene, as well as volatile aldehydes/alcohols, like citral, L-linalool, and nerol [5,28]. Furthermore, these compounds can alter the conformations of polysaccharides, fatty acids, and phospholipid layers [4]. These alterations solidify the cytoplasm and damage the plasticity and cell membrane barriers of pathogens, thereby exerting a direct antibacterial effect. Following glycosylation, phenylpropanoids/benzenes mitigate autotoxicity and induce the activation of the phenylpropanoid metabolic pathway [4]. This induction increases the activities of pathway-related enzymes, facilitating the downstream synthesis of total phenols, flavonoids, lignin, and other resistance-related substances. This process further accelerates lignin deposition, creating a physical barrier analogous to the Casparian strip [29]. This barrier enhances the responses of infected plants to pathogen invasion, thereby exerting an indirect antibacterial effect [30]. As a key enzyme involved in lignin synthesis, *CSE* was analyzed during the post-harvest infection of tomatoes by *Botrytis cinerea*. The expression levels of *SlCSEs* in green mature fruits were less affected by *Botrytis cinerea* infection, compared with red mature fruits; however, in red mature fruits, the expression levels were significantly upregulated in response to infection. Among them, *SlCSE05–07* and *SlCSE10* in red mature fruits exhibited significantly upregulated expression following *Botrytis cinerea* infection. The expression levels of *SlCSE01*, *SlCSE03*, and *SlCSE11* were unaffected by *Botrytis cinerea* infection, potentially due to the ripening stage of the tomatoes. We investigated the mechanism by which 2-ethylfuran influences *SlCSEs* during the post-harvest infection of tomatoes by *Botrytis cinerea*. The results indicated that 24 h post-treatment represents a critical time point for the antibacterial activity of 2-ethylfuran, and many *SlCSEs* displayed upregulated expression during the first 24 h. Additionally, 2-ethylfuran decreased the expression levels of *SlCSEs* at various time points, demonstrating a direct antibacterial effect. Among them, treatment with 7 mL/L of 2-ethylfuran prolonged the duration of increased expression levels of *SlCSE03*, *SlCSE05*, and *SlCSE07*, while reducing the duration of the decreased expression level of *SlCSE06*. It was hypothesized that 2-ethylfuran enhances the responses of *SlCSEs* to disease stress and mitigates damage caused by pathogens. These findings align with the expression patterns of lignin synthesis-related genes in the tomato defense responses triggered by *BcGs1* [8].

Utilizing public databases and our experimental results, *SlCSE06* was identified as a candidate gene that contributes to tomato resistance against *Botrytis cinerea*. Subsequent studies involving the construction of *SlCSE06* overexpression plants revealed a significant increase in lignin accumulation. This finding indicates that *SlCSE06* positively regulates lignin levels in tomato plants. Our results align with previous studies on lignin synthesis involving pear *PbCSE1* [13], *Arabidopsis cse-2* [17], and petunia *ir-PhCSE* [19]. Plant cell walls possess the ability to withstand pathogen invasion. To effectively complete the infection process, pathogens must secrete cell wall-degrading enzymes and employ other mechanisms to compromise the integrity of the cell wall [6,7]. Lignin is a crucial component of the cell wall that significantly contributes to the plant’s defense against pathogenic stress [29]. During *Botrytis cinerea* infection, in comparison to the WT, the OE6 plants significantly increased the expression level of *SlCSE06* and enhanced lignin accumulation, thereby bolstering the overall resilience of tomato plants against *Botrytis cinerea*. This result aligns with the study on cucumber *CsCSE1* [20], which enhances disease resistance by positively regulating additional lignin synthesis-related genes.

## 4. Materials and Methods

### 4.1. Materials

Tomato fruits were obtained from Kanglvnong Agricultural Fruit and Vegetable Co., Ltd. (Quanzhou, China). Tomato fruits of uniform shape and size, free from disease and mechanical damage, were selected for the inoculation test. The wild type (WT) haploid strain *Botrytis cinerea* B05.10 was stored in a laboratory freezer at −80 °C.

The test and transgenic material used was ‘Micro-Tom’ that had been cultivated in the growth chamber of the Fuzhou Smart Seed Industry Technology Innovation Center under the following conditions: a light intensity of 18.5 klx, a light duration of 16 h, a day/night temperature of 28 °C/18 °C, and a humidity of 80%.

### 4.2. Methods

#### 4.2.1. *Botrytis cinerea* Strain Cultivation and Spore Suspension Preparation

In total, 10 μL of WT *Botrytis cinerea*, which had been stored in a 20% glycerol solution, were transferred from the −80 °C freezer. The *Botrytis cinerea* haploid strain B05.10 was then transferred to 90 mm diameter × 20 mm deep dishes containing potato dextrose agar before being incubated at a constant temperature of 22 °C.

After 10 to 14 days of incubation, mycelial blocks were removed and added to 1 mL of sterile water. The samples were then vortexed and shaken to ensure even mixing. Then, they were passed through a sterile filter cloth. Finally, a hemocytometer was used for spore counting. Subsequently, the spore suspension was diluted with sterile water to achieve a concentration of 10^6^ cells/mL.

#### 4.2.2. Tomato Fruit Treatments

The tomato fruits were soaked in a 2% (*v*/*v*) sodium hypochlorite solution for 2 min. After soaking, the fruits were washed with sterile distilled water, and excess water was removed by blotting with sterile filter paper. The tomato fruits were randomly divided into three treatment groups, with each fruit having a small hole (2 mm in diameter and 3 mm deep) at the equator. Each fruit was inoculated in the hole with 10 μL of *Botrytis cinerea*. In the two treatment groups, the *Botrytis cinerea* suspension contained 2-ethylfuran at a concentration of either 7 mL/L or 14 mL/L. The samples were then placed in PET plastic boxes measuring 50 cm × 30 cm × 27 cm. At various storage time points (0, 12, 24, 36, 48, and 72 h), the tomato fruits were randomly selected from each treatment group for image acquisition. Additionally, samples were collected from a radius of 5 mm around the center of the inoculation site and subsequently frozen in liquid nitrogen at −80 °C for future analyses.

#### 4.2.3. Identification and Chromosomal Localization of *SlCSE* Family Members

The *Arabidopsis CSE* family sequences were downloaded from the Arabidopsis Information Resource database (https://www.arabidopsis.org) (accessed on 5 August 2024) [31], and the tomato ITAG5.0 genomic data were downloaded from Phytozome (https://phytozome.jgi.doe.gov/pz/portal.html) (accessed on 5 August 2024) [32]. Two methods were used to identify candidate genes of the *SlCSE* family. First, the TBtools-II software (2.118) [33] was used to analyze the complete genome protein sequences of tomato and the CSE protein sequences of *Arabidopsis*, enabling the identification of candidate genes within the *SlCSE* family. Next, the *CSE* family hidden Markov model (PF12146) was downloaded from Pfam [34] (https://www.ebi.ac.uk/interpro/entry/pfam/#table) (accessed on 5 August 2024) using HMMER [35] (https://www.ebi.ac.uk/Tools/hmmer/) (accessed on 5 August 2024). Then, the Conserved Domain Database (https://ncbi.nlm.nih.gov/cdd) (accessed on 5 August 2024) [36], Pfam [34], and SMART (http://smart.embl-heidelberg.de) (accessed on 5 August 2024) [37] were used to determine whether the selected tomato *CSE* genes contained the complete abhydrolase domain. Finally, after manually removing duplicate and redundant sequences, 11 members of the *SlCSE* family were identified. Based on their positions on the chromosome, these genes were designated as *SlCSE01–11*. The general principles of chromosome localization were visualized using the TBtools-II software.

#### 4.2.4. Physicochemical Property, Structural Characteristic, and Subcellular Localization Predictions for Tomato CSE Proteins

ProtParam (https://web.expasy.org/protparam/) (accessed on 5 August 2024) [38], SOPMA (https://npsa-pbil.ibcp.fr/cgi-bin/npsa_automat.pl?page=npsa_sopma.html) (accessed on 5 August 2024) [39], SWISS-MODEL (https://swissmodel.expasy.org/interactive) (accessed on 5 August 2024) [40], and WoLF PSORT (https://wolfpsort.hgc.jp/) (accessed on 5 August 2024) [41] were used to predict the physicochemical properties, secondary and tertiary structures, and subcellular localizations of the *SlCSE* family members.

#### 4.2.5. *SlCSE* Family Members Phylogenetic Analysis, Gene Structures, and Conserved Motif and Promoter Cis-Acting Element Predictions

The CSE protein sequences for *Arabidopsis*, rice, wheat, pepper, and eggplant were obtained from the Arabidopsis Information Resource, Phytozome, and the Solanaceae Genome (https://solgenomics.sgn.cornell.edu/) (accessed on 5 August 2024) databases [42]. The *CSE*-encoded protein sequences for tomato, *Arabidopsis*, rice, wheat, pepper, and eggplant were analyzed using the MEGA11 software [43], employing both the Neighbor-Joining and Maximum Likelihood methods to construct phylogenetic trees. Default parameters were applied in the program, with 1000 replicates set for analysis. The gene structural analysis was performed using TBtools-II, and the conserved domains and motifs of the SlCSE proteins were identified using the Conserved Domain Database and MEME (https://meme-suite.org/meme/) (accessed on 5 August 2024) [44]. The promoter cis-acting elements of the 2000 bp upstream sequences of *SlCSEs* were analyzed using PlantCare (https://bioinformatics.psb.ugent.be/webtools/plantcare/html/) (accessed on 5 August 2024) [45]. Finally, the data were visualized using the Tree Visualization By One Table (tvBOT) online tool (https://www.chiplot.online/tvbot.html) (accessed on 5 August 2024) [46].

#### 4.2.6. Analysis of *CSE* Collinearity Across Multiple Species and Replication Events Within the *SlCSE* Family Members

The MCScanX [47] software (v1.0.0) was used for the collinearity analysis. TBtools-II facilitated the visualization of the collinearity analysis and the evolution of selective pressure on homologous gene pairs. Additionally, Origin2021 (v9.8.0.200) was employed to visualize the evolution of selective pressure.

#### 4.2.7. Identification of Upstream Transcription Factors Regulating *SlCSEs*

The complete genome of tomato was aligned with the transcriptional binding sites of *Arabidopsis* using the Plant TF Bind Motif Shift plug-in from TBtools-II. Subsequently, upstream transcription factor predictions for *SlCSEs* were conducted using the FIMO [48] online platform (https://meme-suite.org/meme/tools/fimo) (accessed on 5 August 2024) and visualized with ChiPlot (https://www.chiplot.online/) (accessed on 5 August 2024).

#### 4.2.8. Analysis of *SlCSE* Family Members’ Expression Characteristics

Using the ‘MicroTom’ expression database (https://eplant.njau.edu.cn/microTomBase/) (accessed on 5 August 2024) [49] and the Tomato Functional Genome Database (http://ted.bti.cornell.edu/cgi-bin/TFGD/digital/home.cgi) (accessed on 5 August 2024), the transcriptome sequencing results from various tissues and fruits of tomato plants were screened [50]. The transcriptome sequencing data of tomato infected by *Botrytis cinerea* (PRJNA76208537) [51] were downloaded from the NCBI database (https://www.ncbi.nlm.nih.gov/) (accessed on 5 August 2024). TBtools-II was used for quality control and read counting, whereas the transcriptome analysis was conducted using the genomic data of tomato ITAG5.0. Finally, visualization was performed using ChiPlot (https://www.chiplot.online/) (accessed on 5 August 2024).

#### 4.2.9. Analysis of *SlCSE* Family Members’ Expression Profiles in Post-Harvest *Botrytis cinerea*-Infected Tomato After 2-Ethylfuran Treatment

In total, 11 *SlCSE* family members were analyzed using RT-qPCR. Primers were designed using Primer 5.0 (Table 2), with actin serving as the internal reference gene.

The total RNA was extracted from cryopreserved samples using the Vazyme FastPure^®^ Plant Total RNA Isolation Kit (Nanjing, China). Subsequently, the first-strand cDNA was synthesized using the GenStar StarScript III All-in-one RT Mix and a gDNA Removal kit (Beijing, China). RT-qPCR reactions were conducted using the GenStar 2× RealStar Fast SYBR qPCR Mix Kit, with the following components: 10 μL of 2× RealStar Fast SYBR qPCR Mix, 1 μL of cDNA, 0.5 μL of both upstream and downstream primers (10 μM), and 8 μL of ddH_2_O. There were three replicates for each gene per treatment. The detection was performed using a qTOWER^3^ G IVD quantitative gene amplification instrument located in Jena, Germany. The amplification procedure included pre-denaturation at 95 °C for 30 s, followed by 40 cycles of denaturation at 95 °C for 10 s, and annealing at 60 °C for 30 s. The melting curves consisted of 15 s at 95 °C 60 s at 60 °C and 15 s at 95 °C. The experimental results were analyzed using the 2^−ΔΔCT^ method to determine the relative expression of the target gene. Finally, data were visualized using ChiPlot (https://www.chiplot.online/) (accessed on 5 August 2024).

#### 4.2.10. *SlCSE06* Cloning, Overexpression Vector Construction, and Genetic Transformations

The total RNA was extracted from mature leaves of WT ‘MicroTom’ using the Vazyme FastPure^®^ Plant Total RNA Isolation Kit. Subsequently, the first-strand cDNA was synthesized using TIANGEN’s FastKing One-Step Reverse Transcription Kit (Beijing, China). Primers specific to *SlCSE06* were designed using SnapGene (refer to Table 3). The tomato cDNA served as the template for amplifying the target gene. The PCR program consisted of the following steps: pre-denaturation at 95 °C for 3 min, followed by 30 cycles of denaturation at 95 °C for 15 s, annealing at 54 °C for 15 s, and extension at 72 °C for 30 s.

*NcoI* and *BcuI* restriction endonucleases were selected for the double digestion of the p1302 vector plasmid, followed by the insertion of the *SlCSE06* fragment. The p1302-*SlCSE06* recombinant vector was verified from a bacterial culture using PCR and subsequently transformed into Agrobacterium GV3101.

Single colonies containing the p1302-*SlCSE06* recombinant vector were selected and inoculated into 400 μL of LB liquid medium (Kan 100 mg/L, Rif 50 mg/L) before being incubated at 200 rpm for 6 h at 28 °C. Subsequently, 200 μL of the above bacterial solution was transferred to 10 mL of LB liquid medium (Kan 100 mg/L, Rif 50 mg/L) and incubated at 200 rpm for 12 hat 28 °C. Agrobacterium was harvested by centrifugation at 8000 rpm for 10 min. The cells were then rinsed with the infection solution and infected with a hot suspension of Agrobacterium. The optical density (OD600) was subsequently adjusted to approximately 0.5 for later use.

Mature seeds of ‘MicroTom’ were germinated in distilled water at 37 °C for 6 h. They were then soaked in 75% anhydrous ethanol in an ultra-clean workstation for 3 min, followed by three rinses with sterile water. Subsequently, the seeds were sterilized with a 10% sodium hypochlorite solution for 15 min and rinsed again with sterile water three times before being dried. After complete sterilization, the seeds were cultured on 1/2 MS medium. The cotyledons, measuring 0.5 cm × 0.7 cm, were excised after 10 days of culture and transferred to a pre-culture medium for 2 days. The Agrobacterium strain GV3101 containing the recombinant vector p1302-*SlCSE06* was then used to mediate the infection of tomato calli for 15 min, after which the calli were placed in a co-culture medium for 2 days. Following this, the co-cultured calli were transferred to differentiation medium 1 for a screening period of 21 days. Once green growth points emerged, the calli were transferred to differentiation medium 2 for further culturing. When the differentiated seedlings reached approximately 2 cm, the excess callus was removed in an ultra-clean workstation, and the seedlings were subsequently placed in the rooting medium. After root development was complete, the seedlings were transferred to soil for cultivation.

#### 4.2.11. Identification of *SlCSE06*-Overexpressing Plants

*SlCSE06*-overexpression (OE) and WT plants were cultivated until they reached the three-leaf stage, which is characterized by the presence of a central leaf. Leaves from the lower growth points were harvested for total DNA extraction. Specific PCR primers (refer to Table 4) were used to identify positive transgenic tomato plants. The amplified products were assessed using 1% agarose gel electrophoresis to confirm the overexpression of *SlCSE06* in plants. After confirming the transgenic status of *SlCSE06*-overexpressing (OE6) plants, mature leaves from both OE6 and WT were stored at −80 °C. The relative expression level of *SlCSE06* was then measured using the same methodology described in Section 4.2.9.

#### 4.2.12. *SlCSE06* Expression Patterns in Response to *Botrytis cinerea* Infection

Mature leaves of *SlCSE06*-OE plants, matched in age with WT plants, were collected. A 5 μL suspension of *Botrytis cinerea* spores was applied to both sides of each main leaf vein on the upper epidermis. In the control group, 5 μL of sterile water was similarly applied. Each leaf was tested in four replicates. After 24 h of dark treatment, the plants were returned to a normal photoperiod. Throughout the treatment, moisture levels were maintained, with temperatures controlled below 22 °C and relative humidity at or above 90%. This experiment was conducted three times. Phenotypic observations, including measurements of lesion diameters, were made after 5 days. Samples were then collected and stored at −80 °C. The relative expression of *SlCSE06* was assessed using the same method described in Section 4.2.9.

#### 4.2.13. Lignin Content Determination

The sampling method is detailed in Section 4.2.12. The lignin concentration kit (Fuzhou, China), and each treatment was conducted in triplicate.

## 5. Conclusions

**Figure 11:**
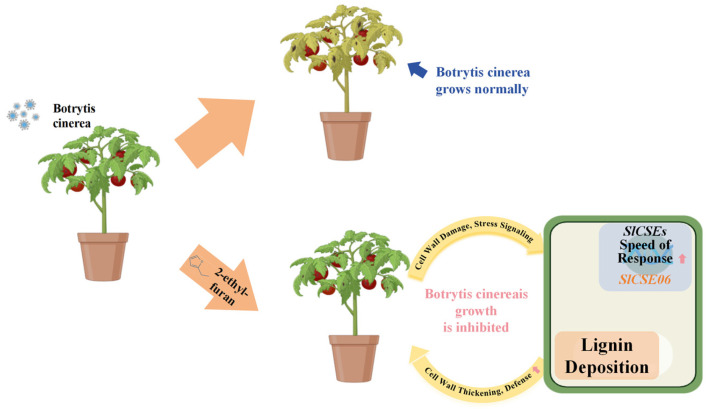
A model illustrating the response mechanisms of *SlCSEs* to post-harvest *Botrytis cinerea* infection-related stress in tomatoes.

Plant VOCs can enhance plant disease-related responses by modulating phenylpropanoid biosynthesis and hormonal signaling pathways. This study aimed to elucidate the synergistic response mechanisms of the *CSE* family and 2-ethylfuran to *Botrytis cinerea* infection in tomatoes. To achieve this, a genome-wide identification and expression analysis of *CSE* genes using the tomato ITAG5.0 genome was conducted. The *Botrytis cinerea* infection-responsive gene, *SlCSE06*, which may be regulated by 2-ethylfuran, was identified. Subsequently, OE6 plants were generated for validation. Ultimately, a mechanistic model demonstrating that 2-ethylfuran activates the disease stress response in tomatoes by modulating the expression of *SlCSE06* was established (Figure 11). A total of 11 *SlCSE* family members were identified in tomato plants. Predictions of cis-acting elements indicated that *SlCSEs* may be involved in multiple stress responses and hormone-related processes. During post-harvest *Botrytis cinerea* infection, the expression levels of *SlCSE05–07* were significantly upregulated, suggesting that these three genes play crucial roles in resistance to this pathogen. Furthermore, the temporal response patterns of *SlCSEs* during *Botrytis cinerea* infection after a 2-ethylfuran treatment revealed peak activities 24 h post-infection, followed by decreased expression levels at other time points. This phenomenon suggests that treatment with 2-ethylfuran enhances the responsiveness of *SlCSEs* and reduces the susceptibility of tomatoes to infection by *Botrytis cinerea*. Based on the expression analysis results, *SlCSE06* was identified as a critical gene involved in the stress responses of tomatoes to *Botrytis cinerea* infection. The construction of OE6 plants demonstrated that under *Botrytis cinerea* infection-related stress, the *SlCSE06* expression level increased, which enhanced lignin deposition and improved resistance to *Botrytis cinerea* infection. These findings elucidated the underlying *Botrytis cinerea* infection response mechanisms and provided theoretical insights for the application of 2-ethylfuran during tomato cultivation.

## Figures and Tables

**Table 1:** Physicochemical properties of *SlCSE* family members.

Gene Name	Gene ID	Number of Amino Acids (aa)	Molecular Weight (MW/Da)	Theoretical pI	Instability Index	Aliphatic Index	GRAV-Y	Signal Peptide	Subcellular Localization	Transmem -Brane Region
*SlCSE01*	Solyc02T000800.1	394	44,478.48	7.65	38.06	90.86	−0.235	No	chlo	outside
*SlCSE02*	Solyc02T000803.1	266	30,136.47	9.17	39.42	92.03	−0.085	No	cyto	outside
*SlCSE03*	Solyc02T001193.1	319	36,048.72	8.4	48.2	88.37	−0.223	No	cyto	outside
*SlCSE04*	Solyc02T002201.1	178	20,378.91	9.05	42.46	92.02	−0.058	No	cyto	outside
*SlCSE05*	Solyc02T002203.1	343	38,511.2	5.8	35.45	91.22	−0.187	No	nucl	outside
*SlCSE06*	Solyc03T003294.1	327	36,292.32	5.91	29.86	71.68	−0.258	No	mito	outside
*SlCSE07*	Solyc04T000407.1	319	36,518.14	6.38	27.44	81.88	−0.36	No	cyto	outside
*SlCSE08*	Solyc05T000443.1	397	44,649.59	8.82	39.93	90.68	−0.253	No	chlo	outside
*SlCSE09*	Solyc05T002712.1	335	37,157.77	6.23	42.32	93.37	−0.016	No	cyto	outside
*SlCSE10*	Solyc08T001425.1	404	45,164.77	8.99	52.75	91.73	−0.096	No	chlo	outside
*SlCSE11*	Solyc09T002257.3	369	41,750.99	8.44	44.97	77.13	−0.389	No	cysk	outside

**Table 2:** RT-qPCR primer sequences.

Genes	Sense Primer (5′ → 3′) F	Anti-Sense Primer (5′ → 3′) R
*SlCSE01* *(Solyc02T000800.1)*	GAGAAGGCAAGTAGTTCGGACAAG	AGGAAGTATGCTCATCCAACCAAG
*SlCSE02* *(Solyc02T000803.1)*	AAAGTGGAAGCAAAGGCGTATCG	TCCTTCATCTTCAATCCAGCATGTG
*SlCSE03* *(Solyc02T001193.1)*	TCACGGTTACTCAGAAGGCTCAC	GCACCACCTAATGACTCACCATAC
*SlCSE04* *(Solyc02T002201.1)*	GTACTGTTCTTCTTGCTCCTCTGTG	ACTGCCGTCTCTCCTAAATCCTG
*SlCSE05* *(Solyc02T002203.1)*	GCCAGAGTTCCGTAATCTACCAAG	AACAGCACCGTTCCAAGAATTAGG
*SlCSE06* *(Solyc03T003294.1)*	GAAGGCAAGCAGTGAGGACAAG	ACAATAGCAACAGCATCGTCAGG
*SlCSE07* *(Solyc04T000407.1)*	CCATCTGTAAGCAAACTCCTCCAC	GCCACACTACAATGTCCGAGAAC
*SlCSE08* *(Solyc05T000443.1)*	TGGAGCAGACGATAGAGTGACAG	AGCACAGTAAGAATCCGATCATCAG
*SlCSE09* *(Solyc05T002712.1)*	GTAGCAGAAGCGAACGAGTTGAG	GTTGTGAGTTACTGAGTGGTGAGAG
*SlCSE10* *(Solyc08T001425.1)*	CTCCTCCGCCTTCGCCATC	CACCGTCATTCTCAACCGTCATATC
*SlCSE11* *(Solyc09T002257.3)*	TATACGAGCAAGCGAGTAGCA	AGCATCACCAGTATTCTCTCCAT
*ACTIN*	GTCCTCTTCCAGCCATCCAT	ACCACTGAGCACAATGTTACCG

**Table 3:** Primer sequences used to used to clone the *SlCSE06* gene.

Primer Name	Sense Primer (5′ → 3′)
p1302-*SlCSE06*-F	GGACTCTTGACCATGGATGGCGTCGGAAGCTCCG
p1302-*SlCSE06*-R	CTTCTCCTTTACTAGTTGCAGAGCCATTGATTTTTGGAC

**Table 4:** Primer sequences used to identify p1302 vectors overexpressing *SlCSE06*.

Primer Name	Sense Primer (5′ → 3′)
p1302 35s-F	GTGGATTGATGTGATATCTCC
p1302 mGFP-R	CTGACAGAAAATTTGTGCCC

## Data Availability

Data are contained within the article.
